# Comparison of the Modified Barthel Index (MBI) Score Trends Among Workers With Stroke Receiving Robotic and Conventional Rehabilitation Therapy

**DOI:** 10.7759/cureus.34207

**Published:** 2023-01-25

**Authors:** Mohd Khairul Anwar Kamdi, Mohd Nazri Shafei, Kamarul Imran Musa, Muhammad Hafiz Hanafi, Mohd Azmi Suliman

**Affiliations:** 1 Community Medicine, Universiti Sains Malaysia School of Medical Sciences, Kota Bharu, MYS; 2 Community Medicine, Universiti Sains Malaysia School of Medical Sciences, Kota Bahru, MYS; 3 Neuroscience, Universiti Sains Malaysia School of Medical Sciences, Kota Bharu, MYS

**Keywords:** barthel index, rehabilitation, workers, robotic therapy, stroke

## Abstract

Introduction: Stroke is one of the top causes of adult-acquired disabilities and the fifth most prominent cause of death worldwide. Working-age populations contribute about 40% of the stroke cases which occur annually in Malaysia. The modified Barthel Index (MBI) score has been used for self-care assessment to determine if stroke patients can meet their fundamental needs. The study was designed to compare the trend of MBI scores of workers who had a stroke and underwent robotic rehabilitation therapy to those who had conventional therapy.

Methodology: A cohort study was conducted among workers who had a stroke in northeastern Malaysia. They were assigned either to undergo robotic or conventional rehabilitation therapy. The robotic therapy is performed three times per day for four weeks. Meanwhile, conventional therapy involved walking exercises five days per week for two weeks. Data were collected for both therapies on the admission, at week 2 and week 4. The MBI, modified Rankin Scale (mRS) and Hospital Anxiety and Depression Scale (HADS) trends were examined one month after the therapies. The R (version 4.2.1) (R Core Team, Vienna, Austria) and RStudio (R Studio PBC, Boston, USA) were applied to perform the descriptive analyses on the respective platforms. Repeated measures of analysis of variance were performed to evaluate the outcomes trend and the effectiveness of the two therapies was also compared.

Results: A total of 54 stroke patients participated in this study of which 30 (55.6%) of them received robotic therapy. The age of the subjects ranged from 24 to 59 years and the majority (74.1%) were male. Stroke outcomes were evaluated using mRS, HADS, and MBI scores. Except for their age, the individuals' characteristics did not significantly differ between those undergoing conventional therapy and those receiving robotic therapy. After four weeks, it was found that the good mRS had increased, whereas the poor mRS had decreased. Comparing the therapy groups, the MBI scores improved significantly with time, although there were no significant differences between the therapy groups. However, the interaction term between the treatment group (p=0.031) and improvements over time was significant (p=0.001), indicating that robotic was more effective than conventional therapy in improving the MBI scores. For HADS score, there was a significant difference between the therapy groups (p=0.001), with those receiving robotic therapy having higher HADS score.

Conclusion: Functional recovery occurs in acute stroke patients when the mean Barthel Index score rises from the baseline (on admission) to week 2 (during therapy) and subsequently on discharge (week 4). Based on these findings, it appears that there was not one therapy superior to the other; nevertheless, robotic therapy may be better tolerated and more effective in certain individuals.

## Introduction

Stroke is a typical neuro-vascular emergency that can be fatal. It is one of the top causes of adult-acquired disabilities and the fifth most prominent cause of death worldwide [1]. It has been expected that the incidence of stroke will continue to rise as the population ages. In addition, breakthroughs in medical technology have reduced the number of stroke-related fatalities, resulting in increased stroke survival with disability. It is estimated that the after-effects of cerebrovascular accidents afflict approximately 1% of the global population [1]. Commonly observed incapacitating signs of a stroke include poor motor control [2], general cognitive deficits, speech production or comprehension difficulties, and altered emotional state [1].

In their prime years, stroke consequences disable individuals physically, psychologically, socially, and economically [3]. About 40% of the 50,000 instances of stroke that occur annually in Malaysia are of working age [4]. As the incidence of stroke increases, Malaysia must be prepared. In 2006, 0.3% of Malaysians were affected by stroke, whereas in 2011, the prevalence rate jumped to 0.7%. Malaysian National Health and Morbidity Surveys from the past demonstrated that there are more strokes among young Malaysians [5].

Independent performance of activities of daily living (ADL) was found to be correlated with return to work. In addition, returning to work before regaining the premorbid stroke condition diminishes the quality of life and burdens the other co-workers [6]. Stroke creates physical and mental disorders that may be regarded as physical disability and might reduce a patient's functionality or ability to perform ADL. Trouble performing ADL enhances the risk of developing depression following stroke [7].

Robotic rehabilitation and assistive technologies have the potential to minimize the stress of physiotherapy personnel and enhance the patient quality of life. The purpose of rehabilitation robotics is to improve rehabilitation using robots. Rehabilitation robotics includes the development of robotic devices to assist varied sensorimotor functions (e.g., arm, hand, leg, ankle), therapeutic training, and patient movement assessment. Robots are utilized as restorative aides, not as assistive gadgets [8].

The Barthel Index of daily living evaluates an individual's ability to perform daily duties. Researchers and clinicians utilize the Barthel index self-care assessment to determine if stroke, neuromuscular, and cancer patients can meet their fundamental needs [9]. The measurement indicates if the patient can recover at home. In clinics and research, the modified Barthel Index (MBI) and modified Rankin Scale (mRS) are the most commonly used instruments [10]. The modified index is designed to identify which ADL a patient can perform without assistance. MBI measures functional outcome of patients after stroke whereas mRS measures the severity of stroke. The mRS takes into account changes in activity and lifestyle after a stroke that do not always match the basic ADL measured by the BartheI Index. The mRS grade seemed to differentiate mild residual disability of stroke survivors sensitively. On the other hand, the MBI provided more detailed information on the functional state of stroke survivors with moderate to severe residual disability. So, both scales were used to measure the severity of the patients and the recovery of the patients.

Depression and anxiety can negatively impact stroke survivors' mental health and quality of life (QoL) [11]. Future therapies must investigate the connections between favorable psychological rates and psychological distress caused by stroke. Depression following a stroke aggravates neurological and psychological disability, negatively affecting ADLs [12]. It was also found that after a stroke, depression also inhibits physical and verbal rehabilitation [13].

The present study aimed to compare the trend of MBI scores of workers who had a stroke and underwent robotic rehabilitation therapy to those who had conventional therapy on admission (baseline), during therapy (week 2), and at discharge from therapy (week 4).

## Materials and methods

Study design and criteria

A prospective cohort study was conducted in April 2021 among workers with stroke who received robotic and conventional rehabilitation therapy at a teaching hospital in northeastern Malaysia. Workers who were diagnosed with stroke or traumatic brain injury for the first time, aged 18-60 as well as able to stand freely or under the supervision and with body size that fits the hybrid assistive limb (HAL) robotic suit were included in the study. Meanwhile, we excluded those with severe cardiovascular or respiratory problems, moderate to severe lower extremity contracture, pacemaker use, and cognitive dysfunction (mini-mental state examination score of 21 out of 30).

For the robotic therapy group, they were having three sessions per day, five days a week, for four weeks. These sessions included physical therapy, occupational therapy, and robotic treatment. A mobile hoist will be used during HAL training to improve the patient's posture and prevent falls. Two physiotherapists were assigned to each patient; one operated the mobile hoist to aid the patient's gait, while the other monitored the HAL and the patient's leg movement. In contrast, the conventional therapy group received two weeks of walking exercises from a physiotherapist. This therapy consisted of one hour of physiotherapy in the morning and one hour of occupational therapy in the evening. The regime of the rehabilitation therapy is the standard care of practice for conventional rehab in the hospital (2 weeks as inpatient and 2 weeks as outpatient). Patient compliance was confirmed using regular reminder and calls. Whereas, patients who received robotic rehabilitation are required to be admitted to undergo rehab for a minimum of 4 weeks. Our study also follows the usual rehab routine for stroke patients that has been routinely practised in Malaysia.

Sample size determination

The sample size is estimated using GLIMMPSE, an online sample size calculator for the general linear mixed model [14]. Using the MBI score as the outcome, the parameter used to estimate the sample size was as follows: (a) Power: 0.8 and Type-1 error: 0.05; (b) Statistical test: Hotelling Lawley Trace test; (c) Number of repeated measures: 3; (d) Hypothesis choice: Interaction (Between x Within); (e) Hypothesis: All mean difference equal zero; (f) Expected MBI mean score [15-16]: Low - Baseline: 40, week 2: 55, week 4: 70, High - Baseline: 35, week 2: 35, week 4: 35; (g) Standard deviation: 30 [16]; (h) Standard deviation ratio: 1:1:1; and (i) Repeated measures correlation: unstructured. A sample of 50 patients for each of the groups was required.

Research tools

Firstly, a designed proforma containing the sociodemographic and clinical information (body mass index, and factors that lead to strokes) of the subjects was filled up. No private or sensitive information was gathered. Each subject was provided with an identification number for further data collection. Secondly, the MBI was applied. The MBI is a straightforward instrument that can evaluate a person's ability to care for themselves and participate in daily activities [17]. The 10-items available in a form of a checklist are as follows: personal hygiene, bathing, feeding, using the toilet, stairs, clothes, bowel and bladder control, chair/bed transfers, and ambulation or wheelchair use. Each activity receives a score based on the support required to complete it. Each category provides an explanation for evaluating the patient's performance and the appropriate number of points. The number of points depends on how much time and assistance the subjects require to execute an everyday task. Lower scores reflected a greater degree of disability. The MBI assessment requires 5-10 min to be completed.

Another tool that was applied in the study was Hospital Anxiety and Depression Scale (HADS). It was developed to measure anxiety and depression in the broader medical community by Zigmond and Snaith [18]. The HADS depends on symptoms that are not somatic to diagnose depression in patients afflicted with severe medical conditions. It has become increasingly popular for application in therapeutic therapy and research settings. Two different subscales of the HADS, each consisting of 14 items, are referred to as the anxiety subscale (HADS-Anxiety) and the depression subscale, respectively (HADS-Depression). On a scale from zero (complete absence) to three (moderately present), the subject assigned a rating to each item. The HADS depression and anxiety components each require a score of 8-9 to be considered clinically significant. It has excellent internal consistency, as shown by a Cronbach's Alpha score of 0.85 [19].

The modified Rankin Score (mRS) was the next tool used in the study. It is a six-point measure of disability. The least severe outcome is positioned at the bottom of a seven-grade scale. Functional independence, mRS scores of 0-2; functional reliance, mRS scores of 3-5; and death, mRS scores of 6, were the three outcome groups [20]. Using mRS, the degree of disability in stroke patients could be determined. The mRS has been analyzed to determine its convergent and content validity, and its inter-rater reliability varies from 0.70 to 0.95 [21]. It also has a significant correlation with the clinical assessments of severity of stroke as well as other disability and outcome endpoints when it is applied correctly.

All subjects were briefed and written consent was obtained before data collection. The data were collected at three different times which were on admission (baseline), during therapy (week 2), and at discharge from therapy (week 4). The MBI score was used to evaluate the patient's disability by the credentialed physiotherapist. The level of disability of stroke patients was evaluated using the mRS. All subjects received two copies of the HADS-Malay version questionnaire. The collected data were maintained securely, with password-protected access.

Statistical analyses

The R-Language and R-Studio were used to analyze the data stored in Microsoft Excel. R (version 4.2.1) (R Core Team, Vienna, Austria) and RStudio (version 2022.07.1) (R Studio PBC, Boston, USA) were utilized to carry out the descriptive analysis on the respective platforms. After analysing the data's distribution, the numerical values were shown using either the mean (standard deviation, SD) or the median (inter-quartile range, IQR). In addition, the categorical data were given in the form of frequency (percentage, %). Repeated measures analysis of variance (RM-ANOVA) was performed to evaluate the outcomes trend and the effectiveness of the two therapies was also compared.

## Results

A total of 54 stroke subjects participated in the study and 30 (55.6%) of them received robotic therapy. The subject's ages ranged from 24 to 59 years old and 40 (74.1%) were male. Among them, 48 (88.9%) had stroke and 6 patients had TBI. There were no significant differences in their characteristics between robotic and conventional therapy, except for age. Subjects receiving conventional therapy were older than those receiving robotic therapy. Table 1 summarizes the distribution of the subjects who participated in this study.

**Table 1:** Comparison of the characteristics of stroke subjects receiving conventional and robotic therapy (n=54). ^a^Welch's t-test; ^b^Pearson's Chi-squared test; ​​​​​​​^c^Fisher's exact test BMI, body mass index

Characteristic		Overall, n = 54	Conventional, n = 24	Robotic, n = 30	p-value
		Mean (SD) / n (%)	Mean (SD) / n (%)	Mean (SD) / n (%)	
Age		44.3 (10.31)	48.5 (9.74)	40.9 (9.63)	0.007^a^
Gender					0.627^b^
	Female	14 (25.9)	7 (29.2)	7 (23.3)	
	Male	40 (74.1)	17 (70.8)	23 (76.7)	
BMI		27.3 (6.21)	27.6 (7.06)	27.0 (5.55)	0.730^a^
Type of Brain Injury					0.682^c^
	Cerebrovascular accident	48 (88.9)	22 (91.7)	26 (86.7)	
	Traumatic brain injury	6 (11.1)	2 (8.3)	4 (13.3)	

In the present study, the outcomes of stroke were evaluated using mRS, HADS, and MBI. An increase in good mRS was observed after four weeks, whereas a fall in poor mRS was observed after the same period. Over four weeks, the MBI demonstrated a significant improvement. Throughout the study duration, neither HADS-Anxiety nor HADS-Depression exhibited significant changes. The mRS, MBI, and HADS measurements were summarized in Tables 2-3.

**Table 2:** The trend of mRS changes among workers with stroke (n=54). mRS, modified Rankin Scale

Score	Time of assessment		
	Baseline, n (%)	Week-2, n (%)	Week-4, n (%)
mRS			
Good	37 (68.5)	42 (77.8)	49 (90.7)
Poor	17 (31.5)	12 (22.2)	5 (9.3)

 

**Table 3:** The adjusted trend of HADS and MBI among workers with stroke (n=54). ^a^Adjusted with Bonferroni adjustment; ^b^Greenhouse-Geisser correction applied HADS, Hospital Anxiety and Depression Scale; MBI, modified Barthel Index

Score	Adjusted Mean (95% CI)^a^			p-value^b^
	Baseline^a^	Week-2^a^	Week-4^a^	
MBI	85.4 (82.34, 88.51)	90.2 (87.07, 93.23)	93.3 (90.23, 96.4)	<0.001
HADS-Anxiety	8.7 (8.04, 9.3)	8.6 (7.96, 9.22)	8.6 (7.98, 9.24)	0.933
HADS-Depression	10.9 (10.24, 11.72)	11.2 (10.47, 11.94)	10.8 (10.08, 11.55)	0.572

Comparing between the treatment groups, the MBI scores improved significantly over time, but there was no significant difference between the treatment groups. Nevertheless, the interaction term between the treatment groups (p-value = 0.031) and changes over time was significant (p-value = 0.001). Improving the MBI scores among those receiving robotic therapy was more effective than in their counterpart. For HADS-Anxiety, there was a significant difference between the treatment groups (p-value = 0.001), in which those in the robotic therapy group had a higher HADS-Anxiety. Otherwise, there were no significant changes over time or interaction terms. HADS-Depression had no significant difference between the treatment groups, changes over time, or interaction terms. The trend and comparison between the treatment group for mRS, HADS, and MBI among the subjects was summarized and shown in Table 4.

**Table 4:** The adjusted trends of HADS and MBI scores between the treatment groups (n = 54). ^a^Adjusted with Bonferroni adjustment; ^b^Greenhouse-Geisser Correction applied HADS, Hospital Anxiety and Depression Scale; MBI, modified Barthel Index

Score		n	Adjusted mean (95% CI)^a^				Effect	p-value^b^
			Baseline	Week-2	Week-4	Overall		
MBI							Between Group	0.638
	Conventional	24	87.8 (83.15, 92.43)	90.3 (85.69, 94.97)	93.1 (88.49, 97.76)	90.4 (87.66, 93.18)	Within Group	<0.001
	Robotic	30	83.5 (79.38, 87.68)	90.0 (85.85, 94.15)	93.5 (89.32, 97.62)	89.0 (86.53, 91.47)	Interaction	0.031
HADS-A							Between Group	<0.001
	Conventional	24	7.6 (6.73, 8.43)	7.3 (6.48, 8.18)	7.4 (6.57, 8.27)	7.4 (6.96, 7.93)	Within Group	0.907
	Robotic	30	9.5 (8.77, 10.29)	9.6 (8.84, 10.36)	9.6 (8.81, 10.33)	9.6 (9.13, 10.00)	Interaction	0.784
HADS-D							Between Group	0.059
	Conventional	24	11.4 (10.29, 12.46)	12.3 (11.16, 13.34)	11.3 (10.16, 12.34)	11.6 (11.00, 12.25)	Within Group	0.483
	Robotic	30	10.7 (9.69, 11.64)	10.4 (9.39, 11.34)	10.5 (9.49, 11.44)	10.5 (9.94, 11.06)	Interaction	0.251

## Discussion

In the present study, we found that those who underwent conventional therapy were older than those who received robotic therapy. Men dominated both receiving robotic and conventional therapy. This is supported by the fact that the proportion of stroke patients was higher among men than women based on a previous study in Malaysia [5, 22]. One of the probable explanations for the finding could be the modifiable factors that raise the risk of stroke owing to a sedentary lifestyle and habits such as smoking and alcohol consumption which can lead to non-communicable diseases (NCDs) such as stroke. Overall, the mean body mass index (BMI) of subjects in both groups falls under the obese category. It is known that obesity increases the chance of developing NCDs, which can lead to stroke.

We found that the overall trend of MBI scores among the subjects was increased from the time of admission to week 2 and week 4 for both robotic and conventional therapy groups. In our study, it is also found that mRS and HADS measurements are the important predictors in the change of MBI scores. In this study, the total mean MBI score improved by approximately 5.5% (from 85.4 to 90.2) and 9.2% (from 85.4 to 93.3) from the time of admission and week 2 and from the time of admission and at discharge (week 4) respectively. It shows that considerable stroke healing occurred over these periods. Early recovery is the consequence of spontaneous neuronal processes [23], and it occurs three months after a stroke [24]. Six months to two years following an acute stroke, the Barthel Index improves, but not considerably [25]. Only within the first year and most importantly within the first six months after the stroke the changes occur. This is consistent with the assumed timeline for functional recovery after a stroke, which suggests that the majority of functional recovery will occur within the first six months after the stroke [26].

When compared to the conventional rehabilitation group, the robotic rehabilitation group demonstrated significant improvement over time in ADLs (11.9%) in the MBI scores. This finding is consistent with the previous study on robotic training in patients with hemiparetic stroke, demonstrating better MBI improvement after the training [27]. Other research on robotic rehabilitation has come to the same conclusion, showing that patients who had a subacute hemiparetic stroke benefited from robotic rehabilitation in terms of their functional recovery in ADLs. In 67 patients who had a subacute stroke, Schwartz et al. (2009) found that after 6 weeks of robotic rehabilitation, clinical ADL assessments improved by 10.8% compared to patients who received routine gait training as part of their physical treatment [28]. Similarly, Chung (2017) observed increased ambulation, mobility, balance, and ADLs (15%) following four sessions of robotic rehabilitation in 41 patients with stroke compared with conventional therapy [29].

Yoon et al. (2022) describe robotic rehabilitation appears to have assisted individuals recovering from a hemiparetic stroke in overcoming their fear of falling and increasing their confidence in their ability to execute ADLs [30]. This is supported by one of the robotic rehabilitation benefits which is creating a more 'natural' interlimb hip-knee-ankle coordinated locomotion and a sufficient number of repetitions (up to 2000 steps) with a safe and accurate gait training regimen [31]. Compared to conventional rehabilitation therapy, intensive locomotor training that is both repetitive and accurate is adequate to allow neuronal plasticity and the related recovery of locomotor function [32]. Additionally, robotic rehabilitation system is beneficial in regulating posture and locomotor functions since it makes only minor adjustments in response to the intensity of gait training and limb synchronization [33].

Concerning anxiety, there was a substantial difference between the therapy groups, with robotic therapy subjects obtaining higher scores. The overall mean score for anxiety in the robotic therapy group was 9.6, compared to 7.4 in the conventional therapy group. According to Bragoni et al. (2013), anxiety is a negative prognostic factor for robotic therapy [34]. Nonetheless, the recovery of an internal locus of control (i.e., patients who believed they were the key causal factor in managing their recovery) was a favorable predictive factor for better outcomes. Therefore, rather than questioning if robotic therapy technologies are helpful, we should determine who will gain the most from robotic therapy.

This study possesses several noteworthy strengths, one of which is an investigation of the impact of robotic rehabilitation therapy on the MBI score among workers who suffer from a stroke. Very few studies have been conducted to investigate the effect of robotic rehabilitation therapy on the ADL which can be assessed using MBI. The outcomes of this study can also provide information to the respective agencies regarding the efficacy of robotic therapy. This information can then be brought to other healthcare facilities for them to adopt robotic rehabilitation as part of their stroke management.

Among the limitations of the present study is the small sample due to the global spread of coronavirus disease 2019 (COVID-19) which has an impact on the data collection process. This is due to the reduction in the number of daily appointments given to the patients. Hence, the number of patients who were required to attend the rehabilitation unit also decreased significantly.

## Conclusions

This innovative study concludes that functional recovery occurs in acute stroke patients when the mean Barthel Index score increases from the time of admission to the discharge for both therapies. Based on these results, it appears that neither therapy was superior to another. Nevertheless, robotic therapy may be more successful in certain individuals. As a result, utilizing robotic therapy in post-stroke patients does not come with any negative consequences or drawbacks. However, examination in a more extensive clinical study is necessary in order to determine the efficacy of this helpful adjunct therapy in the community of people who have had a stroke.
